# Transcriptional regulation of PNPLA3 and its impact on susceptibility to nonalcoholic fatty liver Disease (NAFLD) in humans

**DOI:** 10.18632/aging.101067

**Published:** 2016-10-13

**Authors:** Wanqing Liu, Quentin M Anstee, Xiaoliang Wang, Samer Gawrieh, Eric R. Gamazon, Shaminie Athinarayanan, Yang-Lin Liu, Rebecca Darlay, Heather J Cordell, Ann K Daly, the Investigators FLIP, the Investigators FLPGP, Chris P Day, Naga Chalasani

**Affiliations:** ^1^ Department of Medicinal Chemistry and Molecular Medicine, College of Pharmacy, Purdue University, West Lafayette, IN 47907, USA; ^2^ Liver Research Group, Institute of Cellular Medicine, The Medical School, Newcastle University, Newcastle upon Tyne, UK; ^3^ Department of General Surgery, Shanghai Jiaotong University Affiliated First People's Hospital, Shanghai, P. R. China; ^4^ Division of Gastroenterology and Hepatology, Indiana Fatty Liver Disease Research Group, Indiana University School of Medicine, Indianapolis, IN 46202, USA; ^5^ Section of Genetic Medicine, Department of Medicine, The University of Chicago, Chicago, IL 60627, USA; ^6^ Division of Genetic Medicine, Department of Medicine, Vanderbilt University, Nashville, TN 37235, USA; ^7^ Institute of Genetic Medicine, Newcastle University, Newcastle upon Tyne, UK

**Keywords:** PNPLA3, NAFLD, eQTL, haplotype, gene, fibrosis

## Abstract

The increased expression of *PNPLA3^148M^* leads to hepatosteatosis in mice. This study aims to investigate the genetic control of hepatic *PNPLA3* transcription and to explore its impact on NAFLD risk in humans. Through a locus-wide expression quantitative trait loci (eQTL) mapping in two human liver sample sets, a PNPLA3 intronic SNP, rs139051 A>G was identified as a significant eQTL (*p* = 6.6×10^−8^) influencing *PNPLA3* transcription, with the A allele significantly associated with increased PNPLA3 mRNA. An electrophoresis mobility shift assay further demonstrated that the A allele has enhanced affinity to nuclear proteins than the G allele. The impact of this eQTL on NAFLD risk was further tested in three independent populations. We found that rs139051 did not independently affect the NAFLD risk, whilst rs738409 did not significantly modulate *PNPLA3* transcription but was associated with NAFLD risk. The A-G haplotype associated with higher transcription of the disease-risk rs738409 G allele conferred similar risk for NAFLD compared to the G-G haplotype that possesses a lower transcription level. Our study suggests that the pathogenic role of *PNPLA3^148M^* in NAFLD is independent of the gene transcription in humans, which may be attributed to the high endogenous transcription level of *PNPLA3* gene in human livers.

## INTRODUCTION

Affecting over 30% of the U.S. population, non-alcoholic fatty liver disease (NAFLD) is the most common cause of chronic liver disease [1-3]. NAFLD starts with hepatosteatosis, and can progress to non-alcoholic steatohepatitis (NASH), cirrhosis and hepatocellular carcinoma (HCC) [1-3]. While the etiology of NAFLD still remains incompletely understood, the development of NAFLD has been strongly related to age, energy homeostasis and genetic predisposition [4-6]. In recent years, genome-wide association studies (GWAS) have identified a number of loci conferring risk for NAFLD. Among these, the rs738409 C>G (I148M) variant of the *patatin like phospholipase domain containing 3* (*PNPLA3*) gene located at 22q13 is the most well validated association, influencing degree of steatosis, grade of inflammation, stage of fibrosis and risk of HCC [7-11]. However, the detailed mechanism underlying this genetic association remains incompletely understood [10]. In particular, the biological consequences of this mutation and how it plays a causal role in disease pathogenesis are still unclear. Previous *in vitro* study demonstrated that PNPLA3 plays a role in triglyceride metabolism as a lipase and the 148M alteration disrupts its hydrolysis activity [12]. Other studies indicated that 148M variants may also reduce triglyceride secretion as well as promotes triglyceride synthesis by increasing its lysophosphatidic acid acetyltransferase (LPAAT) activity [13-15]. In further exploring this mechanism, many studies have focused on various animal models. Thus far, it has been consistently shown that neither overexpression (transgenic or Tg mouse) nor loss-of-expression (knock-out or KO mouse) of the wild-type (WT) *Pnpla3* gene induces a NAFLD phenotype in mice (16-18). However, overexpression of the variant form, human *PNPLA3^148M^* (Tg mouse), does lead to hepatosteatosis [18]. This observation is further confirmed in a 148M knock-in (KI) mouse model, in which physiologically regulated background expression of *Pnpla3^148M^* on a normal chow diet does not produce NAFLD, whilst increased *Pnpla3^148M^* expression following consumption of a high-sucrose diet does induce NAFLD [19]. These features parallel the effect of a PNPLA3 catalytic activity-negative mutant, S47A [18, 19]. These studies strongly suggest a “dominant-negative” mechanism underlying the *PNPLA3^148M^* pathogenesis. While the high transcriptional expression of the mutant allele seems to be required for this pathogenic effect in animals, it remains unclear whether there is a similar mechanism in humans. As the level of *PNPLA3* expression in humans is variable, it is conceivable that inter-individual differences in *PNPLA3* gene expression further modify the risk for NAFLD attributed to the 148M variant. Clarifying this mechanism would be essential for establishing therapeutic strategies for NAFLD/NASH. Interestingly, a recent study pointed out that a nonsynonymous common variant rs2294918 (E434K) seemed to be associated with decreased *PNPLA3* transcription, which may modified the effect of rs738409 in NAFLD [20]. However, detailed knowledge about the regulation of hepatic *PNPLA3* gene transcription in human populations is extremely limited. To date, no study has been conducted that fully investigates the inter-individual variability in hepatic *PNPLA3* expression as well as its impact on NAFLD risk.

In order to better understand the transcriptional regulation of *PNPLA3* in human liver, we performed an eQTL (gene expression quantitative trait loci) mapping for *PNPLA3* mRNA expression in human liver tissue samples, using genome-wide transcriptome and genotypic data that we have collected in our previous study [21]. We further studied the relationship between rs738409 and *PNPLA3* gene expression, and investigated the association between significant eQTLs and NAFLD. The combined haplotypic effect of the significant eQTL and rs738409 in conferring NAFLD risk was also analyzed.

## RESULTS

### eQTL mapping

**Figure 1:**
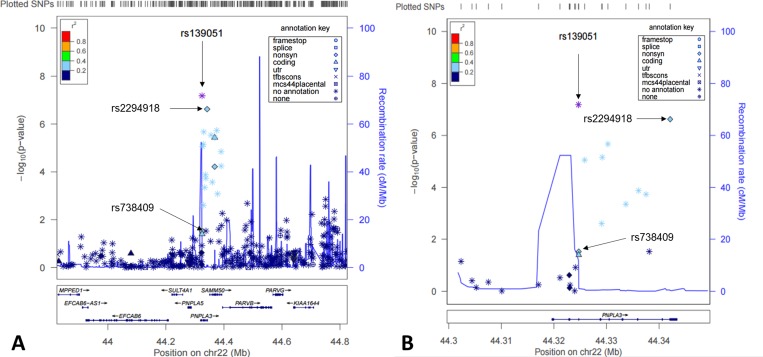
*Cis*-eQTL mapping for *PNPLA3* in human livers Shown here were both statistical significance (−log_10_^*p*^) (left y-axis) and recombination rate (right y-axis) across the *PNPLA3* locus ±1Mb region on Chromosome 22 and centered to rs139051 (**A**) and a zoomed region centered to the PNPLA3 gene region (**B**).

**Figure 2:**
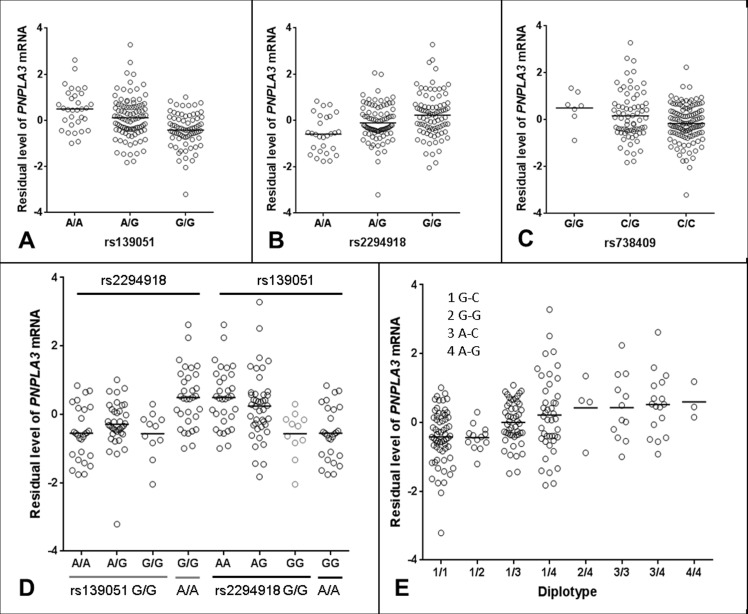
Regulatory role of rs139051 in *PNPLA3* mRNA expression in human liver (**A-C**) Correlations between rs139051 (*p* = 6.6×10^−8^, FDR < 0.05), rs2294918 (*p* = 2.4×10^−7^, FDR < 0.05) and rs738409 (*p* = 0.04, FDR > 0.05) genotypes and *PNPLA3* gene expression. (**D**) The dependence of rs139051 and rs2294918 in association with *PNPLA3* expression. No significant difference in gene expression between rs2294918 genotypes among the rs139051GG homozygous individuals was observed (ANOVA *p* = 0.3), while rs139051 was significantly associated with *PNPLA3* gene expression among the GG homozygous individuals at the rs2294918 locus (ANOVA *p* = 0.006). (**E**) Association between rs139051-rs738409 diplotypes and gene expression. Significant difference was observed when compared 1/1 to 1/3, 1/4, 3/3 or 3/4 (t-test, *p* < 0.003 for all tests), and compared 1/2 to 1/3, 1/4, 3/3 or 3/4 (*p* < 0.05 for all tests). No significant difference between 1/3 and 1/4 or between 3/3 and 3/4 was observed (*p* > 0.05 for all tests). Horizontal bars indicate the mean values of mRNA expression.

Studying genotypic data of 620,900 SNPs as well as imputed genotypic data (in total over 3.9 million SNPs using HapMap release 27 as reference), we focused our analysis on identification of *cis-*acting eQTLs on chromosome 22. We found that the most significant eQTLs were mapped to the *PNPLA3-SAMM50* region (Fig. 1A-B). Two SNPs, rs139051 (51bp upstream of rs738409 in intron 2, and 28bp away from the intron-exon boundary) and rs2294918 (E434K in exon 9) were identified as the most significant *cis*-acting eQTLs for *PNPLA3* transcription [*p* = 6.6×10^−8^ and 2.4×10^−7^, respectively; false discovery rate (FDR) < 0.05 for both] (Fig. 2A-B).

Detailed linkage disequilibrium (LD) analysis of variants across the locus revealed that whilst rs139051 was in low LD with all other significant eQTLs (*r^2^* ≤ 0.50 for all tests), rs2294918 was in high LD with the remaining eQTLs (*r^2^* ≥ 0.80), suggesting an independent role of rs139051. The plotted data indicated that the A allele of rs139051 and G allele of rs2294918 were associated with increased *PNPLA3* gene expression (Fig. 2A, Fig. 2B). In contrast, after correcting for multiple testing, the rs738409 polymorphism was not a significant eQTL for hepatic *PNPLA3* expression (FDR > 0.05) (Fig. 2C).

We further analyzed the inter-dependence of rs139051, rs2294918 and rs738409 in altering *PNPLA3* gene expression. Analysis of the HapMap data (CEU, Northern Europeans in Utah) demonstrated that, although there is low LD among these three SNPs as measured by the *r^2^* values (*r^2^* ≤ 0.26 for all), the D’ values (coefficient of linkage disequilibrium) (D’=0.61, 0.88 and 1 between rs139051 and rs738409; between rs139051 and rs2294918; and between rs738409 and rs2294918, respectively) suggested alleles of these SNPs may share the same haplotype(s). Similar data (low *r^2^* but relatively high D’ values) were observed in both Asian (CHB+JPT, Han Chinese in Beijing and Japanese in Tokyo) and African (YRI, Yoruba in Ibadan, Nigeria) populations (data not shown). The phased HapMap haplotypic data (CEU) suggest that the rs139051A and rs2294918G alleles (both of which are associated with increased *PNPLA3* expression) are almost always co-segregated in the same haplotype (data not shown). Further multivariate haplotypic association with gene expression in liver tissue demonstrated that the association between the rs2294918 G allele and *PNPLA3* expression was actually driven by the rs139051 A allele. In rs139051 G/G homozygous individuals, rs2294918 genotypes did not significantly contribute additional variability in gene expression, which was in contrast with the association between rs139051 genotypes and gene expression among the rs2294918 A/A homozygous individuals (Fig. 2D). Similarly, the association between rs139051-rs738409 diplotypes and the *PNPLA3* mRNA expression also suggested that rs139051 solely contributed to gene expression (Fig. 2E). The independent role of rs139051 in association with gene expression was further verified in a multivariate analysis in which, after controlling for rs139051 genotype, none of the other eQTLs at the locus remained significant (*p >* 0.05 for all tests, data not shown), while after conditioning all other SNPs, rs139051 remained significant (*p* = 8.18×10^−6^). Taken together, these data suggest a potential causal role of rs139051 in regulating hepatic *PNPLA3* transcription.

### Validation of the rs139051 as a significant eQTL

To confirm the association between rs139051 and *PNPLA3* mRNA expression, we used another set of liver samples among which genome-wide transcriptome data has been previously collected in a subset of samples (N = 54) [22]. These samples were collected from bariatric surgery of obese patients in Caucasian origin [22]. We found that again, rs139051 rather than rs738409 was significantly associated with *PNPLA3* transcription (*p* = 0.037 for rs139051; *p* = 0.95 for rs738409). After controlling for age, gender and BMI, this association at rs139051 remained significant (*p* = 0.029).

### Electrophoresis mobility shift assay (EMSA)

**Figure 3:**
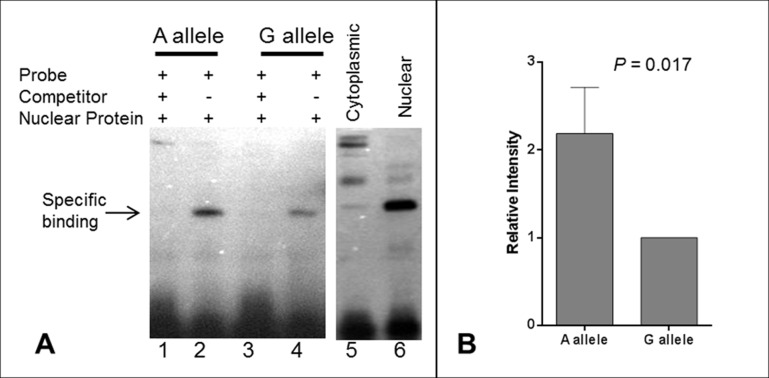
Electrophoretic mobility shift assay (EMSA) for the rs139051 polymorphism (**A**) Representative demonstration of EMSA assay for rs139051 sequence from three independent replications. Relative binding efficiency to nuclear extracts of the HepG2 cells between the A allele and G allele probes were shown (lane 1 and 3). The DNA-protein complex diminished in the reactions with 200X non-labeled competitor probes (lane 2 and 4). The interaction complex was specific to nuclear extract (lane 6) compared to the cytoplasmic extract (lane 5). The binding efficiency between nuclear proteins and the A allele was significantly higher compared to the G allele (*p* = 0.017). Data were shown in mean ± SD (**B**).

In order to further explore the potential causal role of rs139051, we performed an EMSA to examine the potential interaction between the SNP sequence and hepatic nuclear factors. We found that rs139051 exhibited an allele-specific binding to nuclear proteins extracted from the HepG2 cell line, with the rs139051 A allele possessing significantly higher (> 2 fold) affinity to nuclear factor(s) compared to the G allele (*p* = 0.017). This allelic binding specificity to nuclear protein was supported by a much weaker signal in the reaction with proteins extracted from the cytoplasm as compared to the nuclear extracts (Fig. 3A and Fig. 3B), suggesting that this binding is between the DNA and a nuclear-specific protein rather than a cytoplasmic protein that may non-specifically bind to DNA.

### Association between SNPs and NAFLD

To test whether rs139051 or rs2294918 contributes to NAFLD risk, we performed association analyses between rs139051, rs2294918 as well as rs738409 and NAFLD phenotypes in a large cohort of European Caucasian NAFLD patients with histologically characterized disease collected by the FLIP Consortium (N = 949). Consistent with previous studies [23], rs738409 was significantly associated with histological disease severity across key quantitative phenotypes including degree of steatosis (*p* = 1.1×10^−13^), NAFLD Activity Score (NAS, the sum of the scores for steatosis, hepatocyte ballooning degeneration and lobular inflammation and reflects disease activity) (*p* = 1.5×10^−8^) and stage of fibrosis (*p* = 7.7×10^−5^) after adjusting for relevant covariates (age, BMI and presence of type 2 diabetes mellitus). A less strong but statistically significant association between rs139051 and steatosis (*p* = 1.8×10^−4^), steatohepatitis (*p* = 2.1×10^−4^) but not fibrosis (*p* = 0.435) was also observed. Similarly, rs2294918 was also significantly associated with steatosis (*p* = 2.1×10^−5^), steatohepatitis (*p* = 2.8×10^−4^) and fibrosis (*p* = 0.01). However, in the multivariate analysis conditioning on the rs738409 genotype, neither rs139051 nor rs2294918 was significantly associated with these phenotypes (*p* > 0.05 for all tests), suggesting that neither rs139051 nor rs2294918 is independent of rs738409 as a risk factor for NAFLD.

To investigate further whether rs139051 or rs2294918 modifies the effect of rs738409 alleles in contributing to NAFLD risk, we tested associations between the rs139051-rs738409 or rs2294918-rs738409 haplotypes and aforementioned phenotypes. However, analyses examining the relative contribution of each SNP to the overall haplotype effect did not identify a statistically significant independent effect of rs139051 or rs2294918 in the FLIP population. Indeed, the carriage of the A-G haplotype which combines both the high-expression rs139051 A allele and the disease-risk rs738409 G (148M) allele, conferred similar risk for NAFLD phenotypes as compared to the G-G haplotype which contains the rs738409 risk allele, but possesses a relatively lower expression level (*p* = 0.51) (Table 1 for rs139051 and Table 2 for rs2294918).

**Table 1:** Haplotype association with NAFLD histological features and effect of each haplotype on phenotype severity in the FLIP NAFLD Cohort (N = 949)

Histological Phenotype*	Haplotype†	Added Value (95%CI)#	*P*#	P*	Beta‡	*P*‡
**Steatosis (1-4)**	A-G	0	−	9.85 x10^−7^	0.33	3.36×10^−11^
	G-G	−0.08 (−0.31, 0.16)	0.51	3.78 x10^−4^	0.23	0.0014
	A-C	−0.46 (−0.65, −0.28)	9.85 x10^−7^	-	−0.15	0.0108
	G-C	−0.46 (−0.60, −0.32)	1.33 x10^−11^	0.96	−0.23	3.15×10^−8^
**NAS (1-9)**	A-G	0	-	4.81 x10^−4^	0.56	4.27×10^−8^
	G-G	−0.06 (−0.17, 0.05)	0.30	0.05	0.27	0.06
	A-C	−0.16 (−0.25, −0.07)	4.81 x10^−4^	-	−0.15	0.2
	G-C	−0.18 (−0.25, −0.12)	2.66 x10^−8^	0.61	−0.4	2.45×10^−6^
**Fibrosis (1-7)**	A-G	0	-	0.04	0.17	0.01
	G-G	0.11 (−0.04, 0.27)	0.15	9.64 x10^−4^	0.32	0.0013
	A-C	−0.14 (−0.28, −0.008)	0.04	-	−0.15	0.06
	G-C	−0.12 (−0.22, −0.03)	0.0089	0.74	−0.17	0.0092

† Haplotype frequencies across entire NAFLD cohort: A-G 0.2425; G-G 0.09797; A-C 0.1628; G-C 0.4968. Estimated *r^2^* between rs139051 and rs738409 = 0.20.

# Tests for the effect of each haplotype compared to A-G.

* Tests for the effect of each haplotype compared to A-C.

‡ Tests for the effect of each haplotype compared to all other haplotypes combined.

* NAFLD histologically characterized using the semi-quantitative NASH CRN Score (34). The NAFLD Activity Score (NAS) equals the sum of the scores for steatosis, hepatocyte ballooning degeneration and lobular inflammation and reflects disease activity.

**Table 2:** Association between rs2294918-rs738409 haplotypes and NAFLD histolo-gical features and effect of each haplotype on phenotype severity in the FLIP NAFLD Cohort (N = 949)

Histological Phenotype*	Haplotype^†^	Added Value (95%CI)#	*P*#	P*
**Steatosis (1-4)**	G-G	0	-	1.921e-10
	A-G	−0.62 (−1.59, 0.36)	0.24	0.73
	G-C	−0.44 (−0.58, −0.30)	1.921e-10	-
	A-C	−0.45 (−0.58, −0.32)	6.281e-12	0.96
**NAS (1-9)**	G-G	0	-	4.06e-06
	A-G	−0.16 (−0.64, 0.33)	0.53	1
	G-C	−0.16 (−0.22, −0.09)	4.06e-06	-
	A-C	−0.17 (−0.23, −0.10)	1.39e-07	0.77
**Fibrosis (1-7)**	G-G	0	-	0.004
	A-G	0.24 (−0.39, 0.86)	0.46	0.22
	G-C	−0.15 (−0.25, −0.05)	0.0036	-
	A-C	−0.17 (−0.26, −0.07)	0.00036	0.72

† Haplotype frequencies for rs2294918-rs738409 across entire NAFLD cohort: G-G 0.34; A-G 0.005; G-C 0.3; A-C 0.36. Estimated *r^2^* between rs2294918 and rs738409 = 0.28.

# Tests for the effect of each haplotype compared to G-G.

* Tests for the effect of each haplotype compared to G-C.

To further verify these observations in an independent sample, we performed similar analysis in the aforementioned bariatric patient cohort (N = 212) in which quantitative pathological evaluation of NAFLD phenotypes has previously been described [22, 24, 25]. Given the dependence of rs2294918 on rs139051 as a significant eQTL, we did not include rs2294918 in the following analyses. Again, rs738409 but not rs139051 was significantly associated with NAFLD in the univariate analysis (*p* = 2.0×10^−4^ and 0.093, respective-ly), and no significant independent association between rs139051 and NAFLD phenotypes was observed after conditioning on rs738409 and adjusting for age, gender and BMI. Also, no significant difference between A-G and G-G haplotypes in conferring risk for NAFLD or influencing NAFLD histological features was observed (Table 3). In a subset of 54 samples in which the *PNPLA3* mRNA level was measured, we examined if the *PNPLA3* mRNA expression is a direct co-factor modifying the genetic effect of rs738409 on NAFLD. We found that the *PNPLA3* mRNA level was not significantly associated with any of the NAFLD phenotypes (*p* > 0.05 for all tests) (data not shown). The *PNPLA3* mRNA level did not significantly modify the risk of NAFLD conferred by rs738409 (main effect of *PNPLA3* mRNA level on NAFLD *p* = 0.61; interaction between rs738409 and *PNPLA3* mRNA level with respect to NAFLD (*p* = 0.97).

**Table 3:** Haplotype association with NAFLD and NAFLD histological features, effect of each haplotype on phenotype severity, and effect of PNPLA3 expression together with rs738409 in the bariatric liver samples (N = 212)

Histological Phenotype*	Haplotype^†^	Added Value (95%CI)^#^	*P*^#^	P*	Beta^‡^	*P*^‡^	*P^a^*	*P^b^*	*P^c^*
**Steatosis (0-3)**	A-G	0	-	0.0061	0.41	0.0013	0.00017	0.90	0.26
	G-G	0.14 (−0.39, 0.67)	0.60	0.0087	0.63	0.0072			
	A-C	−0.52 (−0.90, −0.15)	0.0061	-	−0.22	0.077			
	G-C	−0.42 (−0.68, −0.16)	0.0020	0.53	−0.21	0.033			
**NAS (0-8)**	A-G	0	-	0.016	0.48	0.0017	0.00012	0.74	0.54
	G-G	0.11 (−0.32, 0.54)	0.63	0.020	0.68	0.015			
	A-C	−0.38 (−0.69, −0.07)	0.016	-	−0.22	0.13			
	G-C	−0.33 (−0.55, −0.12)	0.0029	0.72	−0.25	0.030			
**Fibrosis** **(0-4)**	A-G	0	-	0.53	0.12	0.18	0.01	0.92	0.57
	G-G	0.27 (−0.27, 0.80)	0.33	0.10	0.42	0.010			
	A-C	−0.16 (−0.66, 0.34)	0.53	-	−0.027	0.76			
	G-C	−0.33 (−0.72, 0.07)	0.12	0.51	−0.13	0.065			
**NAFLD**	A-G	0	-	0.064	0.87	0.0043	0.0018	0.61	0.97
	G-G	0.69 (−0.71, 2.10)	0.31	0.019	1.73	0.014			
	A-C	−0.66 (−1.36, 0.04)	0.064	-	−0.17	0.52			
	G-C	−0.82 (−1.38, −0.26)	0.003	0.56	−0.61	0.0052			

† Haplotype frequencies across entire bariatric cohort: A-G 0.188; G-G 0.057; A-C 0.199; G-C 0.556. Estimated *r^2^* between rs139051 and rs738409 = 0.19.

# Tests for the effect of each haplotype compared to A-G.

* Tests for the effect of each haplotype compared to A-C.

‡ Tests for the effect of each haplotype compared to all other haplotypes combined.

* Histological NAFLD phenotypes were characterized using the semi-quantitative NASH CRN Score [34]. The NAFLD Activity Score (NAS) equals the sum of the scores for steatosis, hepatocyte ballooning degeneration and lobular inflammation and reflects disease activity.

a Test for main effect of rs738409;

b Test for main effect of mRNA level;

c Test for Interaction between mRNA level and rs738409.

In order to expand our observations to other ethnic groups, we further tested the genetic association in a Han Chinese NAFLD patient (N = 384) and healthy control (N = 384) population with matched age and gender that are reported in our previous study [26]. After adjusting for age and BMI, both rs738409 and rs139051 were individually associated with sonographically detected NAFLD (*p* = 2.0×10^−4^ and 1.3×10^−3^, respectively). Once again, after controlling for rs738409, rs139051 was no longer associated with NAFLD (*p* = 0.22). In testing the association between rs139051-rs738409 haplotypes and NAFLD, we again found that the A-G and G-G haplotypes confer similar risk of NAFLD (*p* = 0.27) (Table 3).

Given that the rs738409 G allele was consistently demonstrated to be significantly associated with increased risk for NAFLD phenotypes in all three cohorts, the rs738409 C allele was correspondingly significantly associated with reduced risk. We investigated whether rs139051 modifies this protective effect of the rs738409 C allele. Again, no statistical significance was found between the A-C and G-C haplotypes in conferring the reduced risk for NAFLD in all three cohorts (*p* > 0.2) (Table 1, 3-4).

**Table 4:** Association between rs139501-rs738409 haplotypes and NAFLD in the Han Chinese cohort (N = 768, with case : control = 1:1)

Haplotype^†^	Relative OR (95%CI)^#^	*P*^#^	P*	Overall OR^‡^	*P*^‡^
A-G	1.0 (ref)	-	0.018	1.53	1.37×10^−4^
G-G	0.39 (0.07, 2.17)	0.27	0.46	0.49	0.41
A-C	0.73 (0.56, 0.95)	0.018	-	0.92	0.52
G-C	0.63 (0.50, 0.80)	1.01×10^−4^	0.29	0.72	0.002

† Haplotype frequencies across entire population: A-G 0.371; G-C 0.379; A-C 0.245; G-G 0.005. Estimated *r^2^* between rs139051 and rs738409 = 0.35.

# Tests for the effect of each haplotype compared to A-G.

* Tests for the effect of each haplotype compared to A-C.

‡ Tests for the effect of each haplotype compared to all other haplotypes combined.

## DISCUSSION

The causal mechanism of PNPLA3148M for NAFLD remains incompletely understood. While high expression level of the mutant isoform is critical for the induction of the phenotype in animals, it is not clear whether inter-individual difference in PNPLA3 expression in humans would modify the pathogenic effect of the 148M mutant. In this study, we for the first time comprehensively investigated the genetic control of the PNPLA3 transcriptional variability in human livers. We describe a novel genetic variant significantly influencing hepatic mRNA expression of PNPLA3 that, at least in part, explains the recognized inter-individual differences in PNPLA3 transcription in human livers. Capitalizing upon knowledge of this eQTL, we studied whether the pathophysiological effect of the non-synonymous rs738409 (I148M) variant is solely attributed to the amino acid change or whether transcriptional regulation is also as relevant in humans as it appears to be in mice. Our analyses suggest that PNPLA3 gene expression does not significantly modify the risk for NAFLD in humans.

Our data clarifies a number of critical points raised by previous translational studies using animal models but not yet clarified in humans. Studies in animal models have demonstrated that: 1) The pathogenic role of the 148M allele is not via altering the PNPLA3 transcription. Knockin (KI) of the 148M allele into the mouse Pnpla3 gene did not cause a significant change to the gene expression [19]. 2) Variability in wild-type PNPLA3 transcription alone does not contribute to the fatty liver phenotype. The mice models with either knockout PNPLA3 [16, 17] or overexpression of the wild-type PNPLA3 [18] did not lead to fatty liver. 3) An increased transcription of the PNPLA3148M is required for the induction of hepatosteatosis in mice. In the transgenic mouse model created by Li et al., overexpression of the PNPLA3148M mutant form in the liver under normal chow diet feeding successfully induced hepatic steatosis. Meanwhile, in the recent 148M-KI model study, hepatic fat accumulation was produced only when the expression of the Pnpla3148M isoform was induced [19]. 4) There seems to be a strong gene-environment interaction in the PNPLA3148M function in mice. The PNPLA3 transcription can be significantly induced by nutritional stimuli, especially carbohydrates [27-31]. It was shown that in the PNPLA3148M Tg mice, high sucrose-diet feeding as a stimuli for PNPLA3 transcription exacerbates the phenotype [18]. Furthermore, in the KI model, Pnpla3 was up-regulated by increased carbohydrates intake (high-sucrose diet feeding), which leads to fatty liver [19]. In contrast, the interaction between high fat diet and PNPLA3 genotype is less significant. While high fat diet feeding can significantly increase PNPLA3 protein level in the liver, it does not alter the PNPLA3 transcription [27]. There is also no difference in hepatic fat accumulation in mice carrying different genotype at the 148 position [18, 19]. These findings indicate that in mice, the pathogenic role of Pnpla3148M significantly depends on the high level of hepatic expression of this gene, and this effect may be further augmented by environmental factors especially carbohydrate intake.

Our study in humans confirmed some of these observations in mice. First, we show that carriage of the rs738409 G allele does not alter PNPLA3 transcription in humans, suggesting that, as in mice, the effect of the rs738409 variant in humans is more likely related to the functional impact of the amino acid substitution rather than any quantitative effect on gene expression. This assertion is further supported by reports that 148M directly alters the enzymatic activity of PNPLA3 [12, 18]. Second, our data demonstrated that variability in PNPLA3 transcription alone does not contribute to the risk of fatty liver or disease severity. In our samples, the PNPLA3 mRNA level was neither associated with NAFLD nor with the degree of steatosis. Furthermore, we identified that the rs139051 variants as a strong eQTL plays an independent role in regulating PNPLA3 transcription in man. However, after controlling for rs738409, rs139051 was neither significantly associated with NAFLD, nor with disease severity in any of the populations studied.

On the other hand, we also observed significant differences in the pathogenic effect of PNPLA3 between human and mouse. First, our study indicates that the pathogenic function of the PNPLA3148M isoform in humans does not rely on the transcriptional variability. The apparent changes in hepatic PNPLA3 expression due to rs139051 carriage do not modify the effects of rs738409 G-allele carriage in promoting NAFLD and related phenotypes. This is supported by the analysis of associations between rs139051-rs738409 haplotypes and histological features of NAFLD. While the A-G haplotype represents a higher expression level of the disease risk allele than the G-G haplotype, there is no significant difference between these two haplotypes in conferring risk to NAFLD or disease severity (Table 1-3). A similar effect was observed between the A-C and G-C haplotype in conferring reduced risk for NAFLD. Moreover, the multivariate analysis using PNPLA3 mRNA level as either a direct covariate or an interacting effect in the bariatric liver samples did not show a modification for the NAFLD risk attributed to the rs738409 G-allele (Table 2). Taken together these results suggest that the absolute PNPLA3 expression levels has a lesser effect in human NAFLD as compared to that in mice. Second, it seems that the regulatory role of rs139051 remains significant even in the livers of obese patients undergoing bariatric surgery, suggesting that metabolic conditions under obesity do not overtake the genetic effect of rs139051. Moreover, the association between rs139051-rs738409 haplotypes and NAFLD or NAFLD severity was also not modified in the obese population, further suggesting that environment-gene interaction plays a minimal effect on the PNPLA3148M pathogenesis in humans as well.

**Figure 4:**
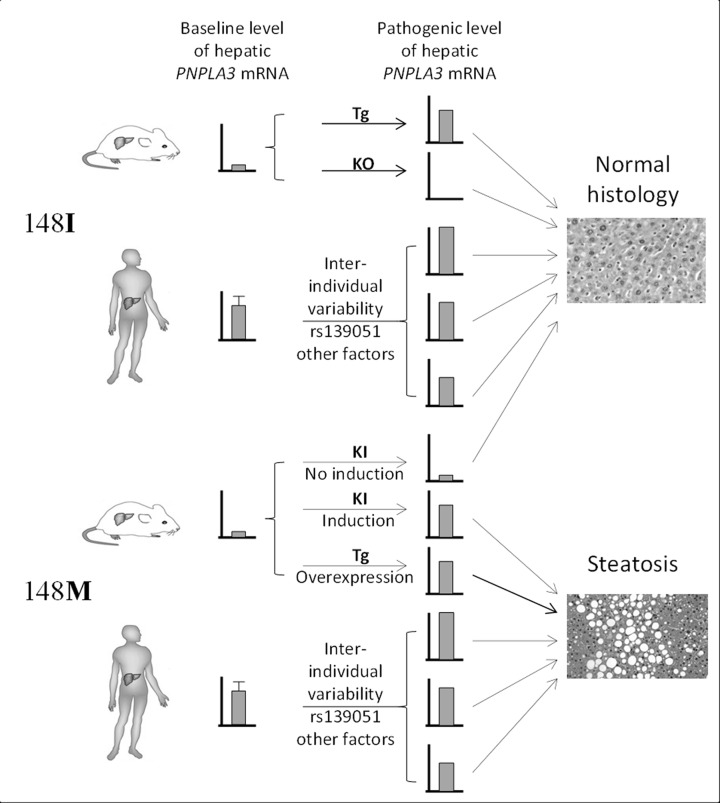
Summary of the difference in the role of gene transcription in *PNPLA3^148M^* pathogenesis between human and mouse Transcriptional variability of 148I isoform does not lead to hepatosteatosis. Very low expression of 148M isoform in mice due to the low baseline *Pnpla3* expression does not induce steatosis as well. However, increased expression of *PNPLA3^148M^* in mice or a higher baseline transcriptional level of *PNPLA3^148M^* in humans leads to steatosis. This highlights the “dominant-negative” effect of the 148M allele in both species. Tg = transgenic, KO = knockout, KI = knockin.

The divergence between our findings in humans and what have observed in animal models may reflect a fundamental difference in disease pathogenesis between species, and highlights the limitations of using animal models in recapitulating human pathophysiology. Given the differences in diet and other environmental factors between human and mouse, it is possible that the two species are under different selection pressure during evolution, which further leads to differential transcriptional regulation of hepatic genes. Indeed, the PNPLA3 gene undergoes a higher baseline hepatic expression in humans than in mice. This has been demonstrated in recent studies that the PNPLA3 mRNA expression is highly expressed in human livers [27], while in normal chow diet-fed wild-type mice, the hepatic Pnpla3 mRNA level is extremely low (in C57BL/6 and LDLR−/− mice) [32] or even undetectable (in SWISS mice) [33]. As a result, the higher baseline expression of PNPLA3 in most human liver may already be sufficient for the pathogenic function of the 148M form, and additional regulation of PNPLA3 in humans by either variant alleles or other environmental factors therefore would not be necessary. Notably, based on the frequency of rs139051-rs738409 haplotypes calculated in Tables 1-4 (see footnotes of the Tables), the A-G haplotype respectively accounts for over 70% and almost 99% of all haplotypes containing the rs738409 G allele in Western populations (71.3% in FLIP and 76.7% in FLPGP cohort) and in Chinese, suggesting that the majority of individuals carrying the rs738409G allele actually also have an increased PNPLA3 transcription level. Furthermore, the discrepancies between the two species may also reflect the distinct dependence on the pathways in lipid homeostasis between the two species. PNPLA3 expression in humans is dominated by the liver followed by skin and adipose tissue [27], while in mice, Pnpla3 has the highest expression in adipose tissue [32], indicating that hepatic PNPLA3 plays a more important role in human lipid metabolism than in mice. Therefore, hepatic lipid homeostasis in humans may be more sensitive to the effect of PNPLA3148M. It is thus very possible that the prevalence of the rs738409 in human population is actually a protective mechanism with which accumulation of lipids in the liver, especially during malnutrition or other environmental conditions, is highly beneficial. Nevertheless, these data together still indicate that a considerable level of hepatic expression of the PNPLA3148 is essential for the induction of fatty liver, though a “pathogenic transcriptional threshold (i.e. the transcriptional level that is sufficient for the expression of the pathogenic effect of 148M)” remains unknown. The similarity and difference in the PNPLA3148 pathogenesis between human and mouse were summarized in Fig 4.

We note that rs2294918 has been associated with PNPLA3 gene expression in human liver and was reported to confer additional risk for NAFLD and NASH independent of rs738409 in a recent study by Donati et al [20]. However, a comprehensive locus-wide eQTL mapping in our study has excluded the causal role of rs2294918 on altering the PNPLA3 transcription. Also, our conditioning analyses have excluded the independent effect of either rs139051 or rs2294918 on disease risk across multiple populations. The discrepancies between Donati's study and our findings could be due to the differences in population that were studied (Italian populations by Donati et al. vs. mixed European or American Caucasians and Chinese in our study), sample size (our study cohort, although substantial, was slightly smaller), or phenotypes to be investigated (NAFLD or NASH for a case-control design in Donati et al. as compared to a quantitative trait analysis on disease severity in our study). Notably, given the low frequency of the G-G haplotype in both Caucasian and Chinese populations as mentioned above, it is possible that the sample size was not sufficient to distinguish the moderate difference in the contribution of disease risks between the A-G and G-G haplotype. These possibilities thus warrant further study of this question in larger cohorts of NAFLD patients that are uniformly phenotyped, and among different ethnic groups.

In conclusion, our study revealed a genetic basis underlying variation in human hepatic PNPLA3 transcription. Using this information, our study further clarified the role of this variation, as well as its impact on the established role of the 148M variant in NAFLD susceptibility. The apparent divergence between our results and what has previously been reported in animal models highlights the complexity underlying the role of the PNPLA3148M variant in the development of NAFLD and potentially offers important new insights into the consequent pathogenic mechanisms both in mice and humans.

## MATERIALS AND METHODS

### Ethical statement

This study consists of samples obtained from several sources and parent studies as described below. Samples were collected with written informed consent obtained from participants with approval of institutional review boards (IRBs) at each participating institution. The Purdue University IRB has approved the use of the liver samples for the purpose of this study (approval number 1202011870). The study was carried out in compliance with the Helsinki Declaration.

### Study populations

FLIP samples: A cohort of 949 unrelated patients of European-Caucasian descent with histologically characterized NAFLD representing the full spectrum of disease from simple steatosis through steatohepatitis to advanced fibrosis and cirrhosis was assembled from centers across Europe as part of a GWAS study by members of the FLIP consortium. The study had the necessary ethical approvals in all recruiting centers and all participants gave informed consent. Alternative diagnoses were excluded, including excess alcohol intake, chronic viral hepatitis (hepatitis B and hepatitis C), autoimmune liver diseases, hereditary hemochroma-tosis, α1-antitrypsin deficiency, Wilson's disease and drug induced liver injury.

Bariatric NAFLD samples: The samples (N = 212) were collected under a study protocol that had been previously reviewed and approved by the Medical College of Wisconsin's Institutional Review Board. Each participating subjected gave a written Informed consent for participation. Subjects were of northern European descent, morbidly obese (BMI ≥ 40 kg/m^2^ or > 35 kg/m^2^ with significant comorbidities) and prior unsuccessful attempts to lose and maintain weight, who underwent bariatric surgery. A protocol intraoperative liver biopsy was performed on all patients for histological phenotyping. Patients with alcohol intake >20 g/d and those with other liver diseases based on positive disease-specific serological tests and suggestive liver histology were excluded. Patients using drugs associated with NAFLD prior to liver biopsy were excluded [22, 24]. An experienced pathologist read the liver biopsy according to the NIH NASH Clinical Research Network working group system [34]. Genome-wide transcriptome analysis in the 54 samples in this study was performed previously [22].

Han Chinese samples: Blood samples for ultrasonography-defined NAFLD patients (N = 384) and healthy controls (N = 384) were collected in the health examination center of Shanghai Jiaotong University Affiliated Hospital (Shanghai, China) based on a previously study approved by the Shanghai Jiaotong University Affiliated Hospital [26]. The NAFLD diagnosis was according to the guideline defined by the Chinese National Consensus Workshop on Nonalcoholic Fatty Liver Disease [35]. Detailed information for this community-based cohort was described previously [26]. Briefly, physical examination, ultrasound screening, medical history review, questionnaire-based survey for diet, smoking and alcohol intake, as well as biochemical laboratory tests were performed. Cases with known causes of steatosis, e.g. heavy alcohol intake (> 20 g/day), the use of medications/herbals known to contribute to hepatic steatosis, as well as hepatitis B and C virus infection were excluded. Patients with high likelihood to have other known liver diseases including autoimmune disease and primary biliary cirrhosis based on aforementioned information were also excluded. The case and control groups were matched for age and gender with both consisted of 229 males and 155 females, as well as a mean age and standard deviation (SD) of 45 ± 13 years.

The study populations were summarized in Table 5.

**Table 5:** Study populations and samples for eQTL mapping and NAFLD susceptibility

Cohorts	Dataset/phenotype	Number of samples	Tests	Ethnicity	Purpose
eQTL Liver	DNA	206	eQTL mapping	Mainly Caucasian with a small number of African American	eQTL discovery
	RNA				
FLIP	DNA	949	Association between SNPs and NAFLD histology	European Caucasian	Phenotypic association discovery
	NAFLD histology				
Bariatric Liver	DNA	212	eQTL confirmation	American Caucasian	eQTL validation Phenotypic association validation
	RNA	54			
	NAFLD histology	212	Association between SNPs and NAFLD histology		
Chinese NAFLD	DNA	384 pairs (case + cont.)	Association between SNPs and NAFLD	Han Chinese	Phenotypic association validation
	Ultrasonographic NAFLD				

### eQTL mapping for PNPLA3

The liver sample set (N = 206) used to perform eQTL mapping has been described in our previous study [21] (Table 5). Briefly, genotyping and transcriptome profiling on these samples were performed on the Illumina Human 610 quad beadchip platform (GPL8887) and the Agilent 4×44 arrays, respectively (GEO accession: GSE28893). Genotype imputation was conducted with IMPUTE2 program [36] after prephasing the genotypes with SHAPEIT [37] using the HAPMAP release 27 as a reference to obtain genotypes for a total of over 3.9 million SNPs. Standard quality control (QC) process was performed by assessing SNP/sample call rates, minor allele frequency (removal of SNPs with frequency < 15%), deviation from Hardy-Weinberg equilibrium (*p* < 0.001), identity-by-decent analysis, sex imputation for each sample, etc., as previously described [21, 38]. Age, gender and the first 3 components of the principal component analysis (PCA) for quantifying the ancestry of samples were included as covariates for eQTL mapping. We also employed the probabilistic estimation of expression residuals (PEER) framework [39] to quantifying unknown hidden factors. To perform the *PNPLA3* gene eQTL mapping, we focused only on the *cis*-acting eQTLs within the ±1Mb region of *PNPLA3* gene. The eQTL mapping was performed using the --assoc function for quantitative traits in PLINK [40], which correlates allele dosage with changes in the trait. False discovery rate (FDR) was used for adjusting the multiple testing [41]. Plotting of the p values of SNPs was conducted using LocusZoom [42].

### Genotyping

Genotyping of rs738409 and rs139051 in DNA samples were performed using Taqman assays according to the instructions provided by the manufacturer (Life Technologies, CA, USA).

### Electrophoresis Mobility Shift Assay (EMSA)

EMSA assay was performed according to our protocol published previously [43]. Briefly, HepG2 cells were cultured in standard conditions and collected for both nuclear and cytoplasmic protein extraction using a NE-PER Nuclear and Cytoplasmic Extraction Kit commercial kit (Thermal Scientific, IL, USA). EMSA was performed with 2.5μg of total nuclear or cytoplasmic extracts using a LightShift Chemi-luminescent EMSA Kit (Thermal Scientific). Biotinylated probe sequences for rs139051 A and G alleles were Biotin/GGGTGCTCTCGCCTATAACTTC TCTCTCCT and Biotin/GGGTGCTCTCGCCT*G*TAAC TTCTCTCTCCT, respectively. Non-biotin labeled probes in a higher concentration (200×) than the labeled probes were used as competitors. EMSA bands were visualized by exposed to the CL-XPosure film (Thermal Scientific) for 3 min. Films were then scanned and the intensity of the band interested was quantified using the ImageJ software [44]. EMSA assays were independently performed three times.

### Haplotype estimation and diplotype assignment

Haplotype frequency between rs139051 and rs738409 among the eQTL mapping population as well as the diplotype for each individual were estimated and assigned by using PHASE [45] under default settings. To plot the data, samples with the diplotype assignment probability lower than 90% were excluded.

### Data analysis

Linkage disequilibrium and haplotype analyses between rs139051, rs738409 and rs2294918 in HapMap samples (CEU, HCB+JPT and YRI) were performed using Haploview 4.2 [46]. Comparisons of gene expression level between genotypes were conducted using one-way ANOVA, and the comparison for allelic binding efficiency in EMSA assays was conducted with unpaired t-test based on the data collected from three independent experiments. Association between diabetes and NAFLD or *PNPLA3* mRNA expression and interaction between polymorphisms and diabetes was examined using linear (for quantitative phenotypes) or logistic (for binary phenotypes) regression analysis within the R package. The genetic association between SNPs or haplotype and NAFLD or NAFLD-related phenotypes (including the effect of adding one SNP to a model that already included the effect of the other SNP) was analysed using the software programs R, PLINK [40] and UNPHASED [47, 48]. The same programs were used for the multivariate test for inter-dependence between rs139051 and the remaining significant eQTLs. Univariate comparisons of gene expression between genotypes/diplotypes were performed using a t-test or ANOVA test. Data plotting was carried out using Graphpad Prism 6.00 (Graphpad software Inc, CA, USA).

## ABBREVIATIONS

eQTL: expression quantitative trait loci
NAFLD: nonalcoholic fatty liver disease
PNPLA3: patatin-like phospholipase domain-containing protein 3
SNP: single nucleotide polymorphism
mRNA: messenger ribosomal nucleic acid
EMSA: electrophoretic mobility shift assay
HCC: hepatocellular carcinoma
GWAS: Genome-wide association studies
NASH: nonalcoholic steatohepatitis
FDR: false discovery rate
NAS: NAFLD Activity Score
ChIP-seq: chromatin immuno-precipitation-sequencing
FLIP: Fatty Liver Inhibition of Progression
